# Perceptions of Telemedicine Use for Specialist Care in Skilled Nursing Facilities

**DOI:** 10.1001/jamanetworkopen.2026.37194

**Published:** 2026-10-02

**Authors:** Gillian K. SteelFisher, Mary G. Findling, Eran N. Ben-Porath, Andrew D. Wilcock, Ateev Mehrotra, David C. Grabowski, Michael L. Barnett

**Affiliations:** 1 Department of Health Policy and Management, Harvard T.H. Chan School of Public Health, Boston, Massachusetts; 2 SSRS, Glen Mills, Pennsylvania; 3 Department of Health Services, Policy and Practice, Brown University School of Public Health, Providence, Rhode Island; 4 Department of Health Care Policy, Harvard Medical School, Boston, Massachusetts

## Abstract

**Importance:**

For long-term residents of skilled nursing facilities (SNFs), greater use of telemedicine for specialist care may improve access to and overall quality of care. Although policy changes during the COVID-19 pandemic facilitated greater use of telemedicine, such use now is rare, and it is unclear why.

**Objective:**

To examine SNF clinical leaders’ perceptions of telemedicine use and explanations of lower use among long-term residents.

**Design, Setting, and Participants:**

This survey study was conducted online and via postal mail from February 7 to July 28, 2025, among directors of nursing of SNFs, the central decision-makers for daily clinical operations. A probability-based sample was drawn from a listing of all 14 084 SNFs in the US, stratified to ensure reach to facilities of different sizes and varying telemedicine utilization.

**Main Outcomes and Measures:**

This study measured the perceptions of key factors associated with quality of care in SNFs. Telemedicine use for specialist care and reasons for telemedicine use and disuse were solicited from SNFs’ directors of nursing. The demographic characteristics of SNFs surveyed were weighted to Centers for Medicare & Medicaid Services benchmarks to reflect the profile of SNFs nationally.

**Results:**

Among 1001 directors of nursing sent invitation letters, 379 (39%) participated in this study. Most directors of nursing whose residents used telemedicine confirmed its proposed advantages: respondents said a top reason for using telemedicine is that it helped less-mobile patients get appointments (77%; 95% CI, 71%-82%) and that it helped improve patients’ quality of care (73%; 95% CI, 67%-78%). Top reasons for not using telemedicine among less-frequent users and nonusers were: telemedicine visits were not frequently offered by specialists (73%; 95% CI, 67%-79%) and telemedicine did not offer higher quality of care over normal practices (62%; 95% CI, 56%-68%). A majority of directors of nursing who use telemedicine (60%; 95% CI, 54%-65%) also reported that telemedicine placed additional time burdens on nursing staff.

**Conclusions and Relevance:**

This survey study found that frontline clinical leaders view specialist telemedicine care as a useful tool for SNFs, particularly for patients with low mobility. However, they reported that telemedicine is offered too infrequently by specialists and adds to nurses’ schedules. Policy and payment reforms should include incentives for specialists to offer telemedicine and reimbursement for nursing staff time.

## Key Points

**Question:**

How do skilled nursing facility (SNF) clinical leaders perceive lower use of telemedicine in the post–COVID-19 era?

**Findings:**

In this national survey of 379 directors of nursing at SNFs, most of those whose residents used telemedicine confirmed the advantages of telemedicine, although they also reported additional time burdens on nursing staff. The top reasons for not using telemedicine among less-frequent users and nonusers were that specialists offered telemedicine only infrequently and there were limited improvements in quality of care over normal practices.

**Meaning:**

The findings suggest that specialist telemedicine care is seen as a useful tool for SNFs, but incentives for specialists to offer telemedicine and reimbursement for nursing staff time are needed.

## Teaser

This survey study assesses how the clinical leaders of skilled nursing facilities (SNFs) perceive long-term residents’ use of telemedicine for specialist care.

## Introduction

More than one million people live as long-term residents in US skilled nursing facilities (SNFs).^1^ Quality of care in these settings is often suboptimal, and among the key challenges is insufficient use of specialist care.^2,3,4,5,6,7,8,9,10,11^ For example, after the transition to long-term care, SNF residents with severe mental illness had a 67% decrease in specialty psychiatry care.^9^ Telemedicine has been widely considered to be a key mechanism for improving quality of care because it allows for more timely access to specialists in both routine and urgent situations. Greater access to specialist care could, in turn, reduce the rate of emergency department (ED) visits and hospital admissions.^3,4,5,6,7,8,12,13,14,15,16,17,18,19^

During the COVID-19 pandemic, policy and payment barriers to the widespread adoption of telemedicine in SNFs were reduced or eliminated.^20,21^ As a result of these changes and concerns about COVID-19 spreading in SNFs, telemedicine use in SNFs increased markedly in early 2020.^13^

However, telemedicine use in SNFs decreased substantially in subsequent years, such that only 2% of SNF visits used telemedicine by 2022.^13^ Reasons for this decrease are not clear, especially because prior research has found that telemedicine for SNF patients, including telemedicine visits for specialist care, has been viewed positively by clinicians and families.^3,6,7,14,21,22^ However, telemedicine may have introduced new logistical challenges in SNFs, such as increased time burden on clinical staff because they must coordinate appointments and be at the bedside to help patients.^4,6,23,24,25^ This increased time burden would be especially challenging in the context of current SNF staffing shortages.^24,26,27^ It could also be that patients or staff were unhappy with the quality of care provided via telemedicine.^3,21,22^ Outside of SNFs, many physicians prefer providing in-person care and feel the quality of telemedicine care is generally inferior to in-person care.^21,28^ Similar considerations might apply in an SNF context.^24^

Given the potential benefits associated with telemedicine in SNFs, this article examines why telemedicine use for specialist care has decreased since the beginning of the COVID-19 public health emergency. Using a national survey among frontline leaders at SNFs, directors of nursing, this study aimed to answer 3 questions: (1) What do directors of nursing see as key challenges to quality of care in SNFs? (2) Do directors of nursing believe that telemedicine offers the proposed benefits of addressing these problems? (3) What are directors of nursing’s views of the reasons for telemedicine’s decrease in their facilities? Insights derived from answers to these questions can inform policies to shape appropriate use of telemedicine in SNFs and the potential benefits associated with increased access to specialty care for vulnerable long-term care residents.

## Methods

### Study Population and Survey Design

We conducted a nationally representative survey of SNFs across the US, online and by postal mail, using a study design following the American Association for Public Opinion Research (AAPOR) best practices for survey research.^29^ The research team developed the survey by starting with qualitative in-depth interviews among nursing home leaders in various clinical and administrative roles to determine which leaders would be able to respond to the final survey with both clinical and administrative perspectives. Ultimately, leaders indicated that study participants should include directors of nursing or equivalent roles, as these key leaders are central decision-makers for daily clinical operations of the SNF and have frontline visibility of the impact of telemedicine on patients and staff. Directors of nursing were asked about in-person and video visits for long-term residents in their SNF. Video visits were defined as those taking place in the facility with a video connection where the clinician could see and hear the resident live on a smartphone, tablet, laptop, or other computer. The survey was designed and analyzed by researchers at Harvard T.H. Chan School of Public Health, and the study was approved by Harvard T.H. Chan School of Public Health’s institutional review board and determined exempt per the Common Rule. Participants provided consent through the survey.

### Data Collection

The survey was fielded from February 7 to July 28, 2025, by SSRS, a national public opinion and research company. Survey participants were drawn from a list of 14 084 SNFs in the US, based on data from the Centers for Medicare & Medicaid Services (CMS).^30^ From this list, we generated a stratified random sample of 3100 SNFs, designed to ensure we included facilities of different sizes and varying telemedicine use, as measured by the number of Medicare fee-for-service part B visits in 2023 and the share of those visits delivered via telemedicine. To obtain accurate contact information, interviewers telephoned the list of 3100 SNFs and successfully obtained information for 1043 directors of nursing, making multiple calls to each facility. There were no major differences between the initial list of 3100 SNFs and the SNFs for whom director of nursing contact information was available with respect to key characteristics of the institutions (eMethods and eTable 2 in Supplement 1). Forty-two of these directors of nursing were selected randomly and sent pilot surveys in fall 2024; they were excluded from the final sample. The remaining 1001 directors of nursing were divided into 2 randomized, replicate samples, which allowed us to evaluate the most effective recruiting strategies in the first round (January 2025) and implement those strategies in a second round (May 2025). Each wave of data collection included an initial invitation via FedEx, which included both a paper survey and return envelope as well as a URL if the respondent wished to participate online. The invitations also included a $75 check as a noncontingent incentive for participation. Nonresponders were sent multiple reminders via postal mail and email.

### Survey Instrument and Measures

Survey questions were developed to address directors of nursing’s perceptions of key factors associated with quality of care, telemedicine, and decreases in telemedicine use. Most questions used 3- and 4-point Likert scales, with full question wording, scales, and responses shown in the eMethods in Supplement 1. Survey content, question wording, response options, and flow were informed by prior surveys on similar topics,^4,6,7,21,24,27,31,32^ as well as preliminary qualitative interviews with directors of nursing and medical directors. After drafting the questionnaire, we conducted cognitive interviews and pilot interviews, making revisions where needed to ensure questions were clear.

### Statistical Analysis

To mitigate the risk of nonresponse bias and ensure the sample reflected the set of SNFs in the US, the sample of respondents was weighted following AAPOR standards to match key characteristics in national data from the CMS Skilled Nursing Facility Quality Reporting Program.^30^ Data were weighted by US Census region, number of beds, 2023 visits, 2023 telemedicine rate, COVID-19 pandemic telemedicine visits, COVID-19 pandemic telemedicine rate, mean number of residents per day, insurance coverage type (Medicare, Medicaid, both), and SNFs’ telemedicine use during the COVID-19 pandemic (high or low) compared to their telemedicine use after the COVID-19 pandemic (high or low). Analyses were conducted in Stata, version 19.0 (Stata Corp), using weighted data. See the eMethods and eTable 1 in Supplement 1 for sampling and weighting details.

## Results

### Study Sample

Of 1001 directors of nursing sent invitation letters, 379 participated in this study (Table 1). The survey completion rate was 39%, following AAPOR standard calculations.^33^ Directors of nursing who participated in this study worked at SNFs that were demographically representative of SNFs nationally (Table 1).

**Table 1.** Demographic Characteristics of Skilled Nursing Facilities Surveyed^a^

Characteristic	No. (N = 379)	Weighted % (95% CI)^b^
Region		
Northeast	61	16.8 (12.9-20.7)
Midwest	118	31.2 (26.3-36.1)
South	138	36.6 (31.5-41.7)
West	62	15.4 (11.6-19.2)
No. of beds		
<100	170	48.0 (42.7-53.3)
100-199	184	46.0 (40.7-51.3)
≥200	25	6.0 (3.5-8.5)
No. of 2023 visits		
1st Quartile (lowest)	95	27.1 (22.4-31.8)
2nd Quartile	95	27.0 (22.3-31.7)
3rd Quartile	92	23.4 (18.9-27.9)
4th Quartile (highest)	97	22.4 (18.0-26.8)
Telemedicine rate (2023)		
1st Quartile (lowest)	97	26.1 (21.5-30.7)
2nd Quartile	81	22.7 (18.3-27.1)
3rd Quartile	105	26.7 (22.0-31.4)
4th Quartile (highest)	96	24.4 (19.9-28.9)
Pandemic visits		
1st Quartile (lowest)	98	27.2 (22.5-31.9)
2nd Quartile	102	26.5 (21.8-31.2)
3rd Quartile	86	24.7 (20.1-29.3)
4th Quartile (highest)	93	21.6 (17.3-25.9)
Mean No. of residents per day		
<51	90	27.1 (22.4-31.8)
51-75	101	26.1 (21.5-30.7)
76-104	96	25.1 (20.5-29.7)
≥105	92	21.7 (17.3-26.1)
Insurance coverage type		
Medicare and Medicaid	354	94.8 (92.5-97.1)
Medicare only	20	3.5 (1.6-5.4)
Medicaid only	5	1.6 (0.3-2.9)
Telemedicine use during and after COVID-19		
High during COVID-19 and low after	101	36.2 (31.1-41.3)
Low during COVID-19 and high after	92	21.9 (17.5-26.3)
High during COVID-19 and high after	106	27.0 (22.3-31.7)
Low during COVID-19 and Low after	80	14.9 (11.1-18.7)
Pandemic telemedicine rate		
1st Quartile (lowest)	81	19.2 (15.0-23.4)
2nd Quartile	84	21.6 (17.3-25.9)
3rd Quartile	108	26.9 (22.2-31.6)
4th Quartile (highest)	106	32.4 (27.5-37.3)

^a^ Source: authors’ analysis of data from a 2025 nationally representative online and paper survey of 379 directors of nursing at US skilled nursing facilities.

^b^ Estimates may not add up exactly due to rounding.

### Perceptions of Challenges to Quality of Care in SNFs

We first asked directors of nursing about 8 key challenges they may face in quality care in their facilities (Table 2). A majority recognized 6 challenges in their care settings, including 2 hypothesized to be addressed by telemedicine (Table 2): 80% (95% CI, 75%-84%) said there were difficulties accessing specialists in a timely way, including 22% (95% CI, 18%-27%) saying it was a major challenge and 58% (95% CI, 53%-63%) saying it was a minor challenge. More than half of the directors of nursing (61%; 95% CI, 55%-66%) said there were challenges with long-term residents being sent to the ED inappropriately, including 11% (95% CI, 8%-14%) saying it was a major challenge and 50% (95% CI, 45%-55%) saying it was a minor challenge. Even greater numbers of respondents reported challenges with restrictions associated with insurance (88%; 95% CI, 84%-91%; major challenge: 39%; 95% CI, 34%-45%; minor challenge: 49%; 95% CI, 43%-54%), nursing staffing shortages (79%; 95% CI, 74%-83%; major challenge: 39%; 95% CI, 34%-44%; minor challenge: 40%; 95% CI, 35%-45%) and nursing aide shortages (81%; 95% CI, 77%-85%; major challenge: 41%; 95% CI, 36%-46%; minor challenge: 41%; 95% CI, 36%-46%), and challenges with non–direct care staffing shortages, such as housekeeping and dietary services (66%; 95% CI, 61%-70%; major challenge: 15%; 95% CI, 12%-19%; minor challenge: 50%; 95% CI, 45%-56%).

**Table 2.** Perceptions of Challenges to Quality of Care for Long-Term Residents Among Directors of Nursing at Skilled Nursing Facilities (N = 379)^a^

Challenge faced by your facility in delivering quality care to long-term residents	Weighted % (95% CI)^b^			
	A challenge (net)	Challenge		Not a challenge
		Major	Minor	
Insurance and managed care–related restrictions	88 (84-91)	39 (34-45)	49 (43-54)	10 (7-14)
Nurse aide staffing shortages	81 (77-85)	41 (36-46)	41 (36-46)	16 (13-20)
Difficulties accessing specialists in a timely way	80 (75-84)	22 (18-27)	58 (53-63)	17 (14-22)
Nursing (registered nurse and licensed practical nurse) staffing shortages	79 (74-83)	39 (34-44)	40 (35-45)	19 (15-23)
Nondirect care staffing shortages (eg, housekeeping, dietary services)	66 (61-70)	15 (12-19)	50 (45-56)	31 (27-37)
Long-term residents being sent to the emergency department inappropriately	61 (55-66)	11 (8-14)	50 (45-55)	37 (32-42)
Difficulties contacting covering clinicians after hours	45 (40-50)	4 (3-7)	41 (36-46)	51 (47-57)
Difficulties accessing primary care clinicians in a timely way	24 (20-29)	2 (1-4)	22 (18-27)	73 (68-77)

^a^ Source: authors’ analysis of data from a 2025 nationally representative online and paper survey of 379 directors of nursing at US skilled nursing facilities.

^b^ Blank or missing responses are included in the total but are not reported. Estimates may not add up exactly due to rounding.

Directors of nursing reported a marked reduction in telemedicine use for specialists in the past year compared with the peak of the COVID-19 pandemic (eMethods in Supplement 1). Fewer than one-third (28%; 95% CI, 23%-33%) reported that long-term residents used video-based visits more than rarely in the past year, down from 79% (95% CI, 74%-83%) during the peak of the COVID-19 pandemic. About half of directors of nursing (51%; 95% CI, 46%-57%) reported using telemedicine rarely in the past year.

### Views on Telemedicine’s Association With Care

Among SNFs that used telemedicine with specialists for long-term care residents in the past year (302 of 379), directors of nursing reported moderately positive views of telemedicine’s ability to improve access and quality (Table 3). A slight majority (52%; 95% CI, 46%-58%) said that telemedicine has improved access to specialists a lot or a moderate amount for long-term residents with limited mobility or trouble traveling for cognitive reasons, and 33% (95% CI, 28%-39%) said telemedicine has improved access to specialists for long-term residents who do not have such limitations. A total of 31% of directors of nursing (95% CI, 26%-37%) said telemedicine improved access to specialists for long-term residents in an urgent clinic situation. When asked about sustaining the quality of care, a majority of directors of nursing reported that on average, long-term residents’ telemedicine visits with specialists provided about the same quality of care (55%; 95% CI, 49%-60%) or better (5%; 95% CI, 3%-8%) than what can be done in person, although 39% (95% CI, 22%-55%) said the quality was worse.

**Table 3.** Views on Telemedicine’s Ability to Improve Care for Long-Term Residents, Among Directors of Nursing at Skilled Nursing Facilities Who Have Had Specialist Telemedicine Visits in the Past Year (n = 302)^a^

Reported views	Weighted % (95% CI)^b^					
	A lot or moderate amount (net)	A lot	A moderate amount	A little or not at all (net)	A little	Not at all
Video-based visits with specialists have improved ________ in your facility a lot, a moderate amount, a little, or not at all						
Access to specialists for long-term residents who have limited mobility or trouble traveling for cognitive reasons	52 (46-58)	20 (16-25)	32 (26-37)	47 (41-53)	33 (28-39)	14 (10-18)
Access to specialists for long-term residents who do not have limited mobility or trouble traveling for cognitive reasons	33 (28-39)	8 (5-11)	25 (21-31)	65 (59-70)	39 (34-45)	25 (20-31)
Access to specialists for long-term residents in an urgent clinical situation	31 (26-37)	12 (8-16)	20 (16-25)	66 (60-71)	29 (24-35)	37 (31-43)
Compared with in-person visits, long-term residents’ video-based visits with specialists placed ____ of additional time burden on nursing staff	60 (54-65)	16 (12-20)	44 (38-50)	37 (31-43)	27 (22-32)	10 (7-14)

^a^ Source: authors’ analysis of data from a 2025 nationally representative online and paper survey of 379 directors of nursing at US skilled nursing facilities. Questions were asked only among facilities that reported using any video-based telemedicine visits with specialists in the past year (n = 302).

^b^ Blank or missing responses are included in the total but are not reported. Estimates may not add up exactly due to rounding.

Among SNFs that used telemedicine with specialists for long-term care residents in the past year (n = 302), a majority of directors of nursing (60%; 95% CI, 54%-65%) reported that long-term residents’ video-based visits with specialists placed at least a moderate amount of added time burden on their nursing staff compared with in-person visits (Table 3).

### Reported Reasons for Telemedicine Use

Among SNFs that used telemedicine with specialists for long-term care residents in the past year (n = 302), the most-cited reasons for use included that it improved access and quality of care (Table 4). Specifically, 77% (95% CI, 71%-82%) reported that telemedicine helped prevent negative outcomes for less-mobile patients with difficulty getting to appointments, and that it helped improve the quality of care for patients at the moment (73%; 95% CI, 67%-78%). A majority of directors of nursing also cited specialists’ (66%; 95% CI, 60%-71%), patients’ (59%; 95% CI, 53%-65%), and families’ (53%; 95% CI, 47%-58%) preferences for telemedicine over in-person visits; that telemedicine helped improve communication among the care team (54%; 95% CI, 48%-60%); and that it helped prevent hospital admissions (53%; 95% CI, 47%-59%) and ED visits (52%; 95% CI, 46%-58%).

**Table 4.** Reported Reasons for Telemedicine Use and Disuse for Long-Term Residents, Among Directors of Nursing at Skilled Nursing Facilities^a^

Reported reason	Weighted % (95% CI)^b^			
	A reason (net)	Reason		Not a reason
		Major	Minor	
Reasons for telemedicine specialist visits among directors of nursing whose residents have had specialist telemedicine visits in the past year (n = 302)^c^				
Helped prevent negative outcomes for less-mobile patients (those with difficulty getting to and from appointments)	77 (71-82)	45 (40-51)	32 (26-37)	20 (16-25)
Helped improve quality of care for the patient at the moment	73 (67-78)	34 (29-40)	39 (33-45)	24 (20-30)
Were sometimes preferred by specialists over in-person visits	66 (60-71)	26 (21-31)	40 (35-46)	31 (26-37)
Were sometimes preferred by long-term residents over in-person visits	59 (53-65)	23 (18-28)	36 (31-42)	38 (33-44)
Helped improve communication between clinicians and other members of the care team	54 (48-60)	15 (11-19)	39 (34-45)	43 (37-49)
Helped prevent admissions to the hospital	53 (47-59)	23 (18-28)	30 (25-36)	44 (38-50)
Were sometimes preferred by family members of long-term residents over in-person visits	53 (47-58)	14 (10-18)	39 (33-45)	45 (39-51)
Helped prevent emergency department visits	52 (46-58)	22 (17-27)	31 (25-36)	44 (38-50)
Helped improve access to specialists after hours	45 (39-51)	19 (14-24)	27 (22-32)	51 (46-57)
Were sometimes preferred by skilled nursing facility staff over in-person visits	38 (33-44)	11 (8-16)	27 (22-33)	59 (53-65)
Became a routine part of our practice	28 (23-34)	9 (6-13)	20 (16-25)	68 (63-74)
Reasons for telemedicine disuse among directors of nursing who have rarely or never had specialist telemedicine visits in the past year (n = 261)^d^				
Not frequently offered by specialists	73 (67-79)	48 (41-54)	26 (20-32)	25 (20-31)
Did not offer much benefit in terms of quality of care over normal practices	62 (56-68)	32 (26-28)	31 (25-37)	35 (29-41)
Did not seem to reduce emergency department visits or hospital admissions	50 (44-56)	20 (15-25)	30 (35-37)	47 (41-54)
Took too much nurse and staff time	49 (43-56)	24 (19-30)	26 (21-32)	48 (42-55)
Not well liked by long-term residents	49 (42-55)	23 (18-29)	26 (21-32)	49 (42-55)
Difficult to set up technology	46 (40-52)	19 (14-24)	27 (22-33)	52 (46-58)
Not well liked by family members of long-term residents	45 (38-51)	17 (13-23)	27 (22-33)	53 (46-59)
Not well liked by staff and nurses	41 (35-48)	15 (11-20)	26 (21-32)	57 (50-63)
Not well liked by specialists	40 (34-46)	17 (13-23)	23 (18-28)	57 (51-63)

^a^ Source: authors’ analysis of data from a 2025 nationally representative online and paper survey of 379 directors of nursing at US skilled nursing facilities.

^b^ Blank or missing responses are included in the total but are not reported. Estimates may not add up exactly due to rounding.

^c^ Questions were asked only among facilities that reported using any video-based telemedicine visits with specialists in the past year (n = 302).

^d^ Questions about disuse were asked only among facilities that reported rarely or never using video-based telemedicine visits with specialists in the past year (n = 261).

### Reported Reasons for Not Using Telemedicine More

Among SNFs that rarely or never used telemedicine for specialist care in the past year (n = 261), specialists’ preferences played a large role in lower use, as did the belief that telemedicine had a limited association with quality-related challenges facing SNFs (Table 4). Specifically, a majority of directors of nursing at facilities that infrequently or never used telemedicine said telemedicine visits were not frequently offered by specialists (73%; 95% CI, 67%-79%) and telemedicine did not offer much benefit in terms of quality of care over normal practices (62%; 95% CI, 56%-68%). Approximately half said telemedicine did not seem to reduce ED visits or hospital admissions (50%; 95% CI, 44%-56%), telemedicine took too much staff and nurse time (49%; 95% CI, 43%-56%), and that it was not well liked by long-term residents (49%; 95% CI, 42%-55%). At least 4 in 10 said the technology needed for telemedicine was difficult to set up (46%; 95% CI, 40%-52%) and it was not well-liked by residents’ family members (45%; 95% CI, 38%-51%), staff (41%; 95% CI, 35%-48%), or specialists (40%; 95% CI, 34%-46%).

## Discussion

Despite the potential for telemedicine to improve access to specialty care in SNFs, it has rarely been used since early in the COVID-19 pandemic.^13^ Results of this national survey of frontline nursing leaders provide key insights as to the reasons why.

First, perceived quality does not appear to be the major reason for low telemedicine use for specialist care in SNFs. Most directors of nursing felt that the quality of care achieved in a specialist telemedicine visit was at least equivalent to in-person care, and perceived improvement to care quality overall was a major motivator for telemedicine use. Furthermore, they said telemedicine was effective in increasing access to specialist care, particularly for less-mobile patients, while noting that access to specialists and inappropriate ED use were key barriers to higher-quality care for their organizations. Instead, the major reason for low telemedicine use appears to be that outside specialists did not frequently offer telemedicine. Secondarily, telemedicine visits added time burdens to a setting already struggling with staffing shortages. Although that was not a barrier for some SNFs, it was a point of concern for others that were infrequent users or nonusers of telemedicine. Finally, many infrequent users and nonusers also suggested that the technology was simply not well-liked by residents, staff, or specialists. This combination of findings offers insights for policy and practice that could promote more effective use of virtual care in nursing homes.

To meet the sustained need for specialist care via telemedicine in the post–COVID-19 US health care system, policy and payment innovations are needed to address the fact that fewer specialists offer telemedicine. It is not clear how much of specialists’ unwillingness to offer telemedicine reflects an industry-wide decline in virtual offerings or a specific reluctance to care virtually for nursing home residents.^3,4,13,34^ Given that 80% of directors of nursing in this survey reported that specialist access was a challenge to delivering quality care, it is very plausible that specialists have low incentives to care for this complex and difficult-to-reach population, regardless of telemedicine availability. This could be compounded by preferences for providing in-person care.^21,35^ Although major payment, policy, and regulatory barriers to telemedicine were eased during the COVID-19 pandemic,^20,21^ additional policies to provide incentives for clinicians could motivate specialists to offer this mode of care. Furthermore, complementary policies to hold nursing homes accountable for appropriate specialty care delivery, including the use of telemedicine, are also needed.

Second, results suggest that policies are needed to address the time burden on SNF staff within a system where there is already a staff shortage.^24,25,26,27^ In this study, the share of directors of nursing facing major challenges with nursing and aide staff shortages outweighed those expressing major challenges in accessing specialists. For SNFs in similar situations, the barriers to implementation may be too great for them to use telemedicine at this time.^36^ However, for those interested in using telemedicine in their facilities, although the technological challenges can be cumbersome and complex,^25^ the main challenges reported in this study lay in the already overstretched nursing workforce and difficulties managing any additional tasks beyond what is absolutely necessary. Taken together with prior research, this is likely a factor when directors of nursing report that clinical staff do not like telemedicine visits.^24,25,26,27^ Overcoming this barrier is likely related to the policy challenges already being widely discussed about how to best improve staffing overall in the nursing home industry.^37^ The Biden administration proposed nursing home staff minimums that were hotly debated, but these standards were repealed under the Trump administration.^38^ Without additional policy movement to bolster the nursing home workforce, this barrier is unlikely to improve soon.

Future research may incorporate direct care staff and a broad range of clinicians who use telemedicine in caring for their patients to gain their perspectives and insights. In addition, lessons learned from telemedicine innovations in Veterans’ Affairs Community Living Centers, such as technology support and protected staff time for participation, may provide useful guidance for clinical leaders seeking to implement this care in their own facilities.^39,40^

As such policies are developed, it may be useful to consider approaches that encourage telemedicine use in settings where it is most needed. Prior to the COVID-19 pandemic, policy was originally designed to make telemedicine available for the most remote facilities, but adoption was practically nil.^13,20^ Findings here suggest that a more sophisticated policy approach would include more focused incentives for facilities with poor specialty care access, even in a broader range of geographies, to enable more appropriate delivery of specialty care.

### Limitations

This study has some limitations, including those common in surveys. Participating organizations may differ from the national population of SNFs, and weighting does not eliminate the possibility that nonresponders differ meaningfully from responders in ways that are not correlated with measured demographic characteristics. It is also possible that those who felt more strongly about telemedicine (positively or negatively) were more likely to respond. We use self-reported data, and respondents may have felt indirect pressure to report positive views of their organizations. We conducted secondary analyses and sensitivity analyses to ensure the results were robust, but the sample size limited us from examining more complex associations within these data.

## Conclusions

In this nationally representative survey study, we found that highly informed frontline clinical leaders endorsed the idea that long-term residents of SNFs, particularly those with low mobility, benefit from access to specialist care via telemedicine. Although many barriers to providing telemedicine in nursing homes exist, the most commonly reported barrier in our survey was external clinicians not offering the option. The infrastructure expanded through the COVID-19 pandemic to offer telemedicine widely in SNFs, but new policy and payment reforms are needed for this care model to thrive in the post–COVID-19 world. In particular, changes should aim to incentivize more specialists to offer this mode of care, and to enable more nursing staff to facilitate telemedicine care without substantially adding to their administrative burden.

## Notes

Supplement 1.
eMethods.
**eTable 1.** Demographic Characteristics of Skilled Nursing Facilities Surveyed and Comparison Between Sample and National Benchmarks for Key Demographics **eTable 2.** Comparison Between Invited Sample and Eligible Sample for Key Demographics

Supplement 2. Data Sharing Statement
