# Supplementary material for: Perceptions of Telemedicine Use for Specialist Care in Skilled Nursing Facilities
Source: JAMA Netw Open. 2026 Oct 2;9(10):e2637194. doi: 10.1001/jamanetworkopen.2026.37194 (PMC13633543; doi:10.1001/jamanetworkopen.2026.37194)
Supplement: Supplementary file 1 [file jamanetwopen-e2637194-s001.pdf]

Supplementary Online Content

SteelFisher GK, Findling MG, Ben-Porath EN, et al. Perceptions of telemedicine use for specialist care in skilled nursing facilities. *JAMA Netw Open.* 2026;9(10):e2637194. doi:10.1001/jamanetworkopen.2026.37194

eMethods.

eTable 1. Demographic Characteristics of Skilled Nursing Facilities Surveyed and Comparison Between Sample and National Benchmarks for Key Demographics

eTable 2. Comparison Between Invited Sample and Eligible Sample for Key Demographics

This supplementary material has been provided by the authors to give readers additional information about their work.

© 2026 SteelFisher GK et al. *JAMA Network Open.*

eMethods.
Survey Question Wording

(Reported among all DONs, n=379)
QS1. Which of the following challenges, if any, does your facility face in delivering quality care to long-term residents? Please indicate whether each is a major challenge, a minor challenge or not a challenge at all.

	A challenge (NET)	Major challenge	Minor challenge	Not a challenge at all	Blank/ Missing
a. Difficulties accessing specialists in a timely way	**80**	22	58	17	3
b. Difficulties accessing primary care clinicians in a timely way	**24**	2	22	73	3
c. Difficulties getting a hold of covering clinicians after hours	**45**	4	41	52	3
d. Nursing (RN and LPN) staffing shortages	**79**	39	40	19	2
e. Nurse aide staffing shortages	**81**	41	41	16	2
f. Non-direct care staffing shortages (e.g., housekeeping, dietary)	**66**	15	50	32	2
g. Long-term residents being sent to the emergency room inappropriately	**61**	11	50	37	2
h. Insurance and managed care related restrictions	**88**	39	49	10	2

© 2026 SteelFisher GK et al. *JAMA Network Open.*

ACCESS

(Only asked of those who have had any video-based visits with specialists in the past year, n=302)
QS2. How much, if at all, would you say video-based visits with specialists improved each of the following in your facility?

	**A lot/A moderate amount (NET)**	**A lot**	**A moderate amount**	**A little/ Not at all (NET)**	**A little**	**Not at all**	**Blank/ Missing**
a. Access to specialist for long-term residents who have limited mobility or trouble traveling for cognitive reasons	**52**	20	32	**47**	33	14	2
b. Access to specialists for long-term residents who don't have limited mobility or trouble traveling for cognitive reasons	**33**	8	25	**65**	39	25	2
c. Access to specialists for long-term residents in an urgent clinical situation	**31**	12	20	**66**	29	37	3
d. How often long-term residents' family members are able to attend specialist appointments	**23**	3	19	**74**	52	22	3

SUSTAINING QUALITY OF CARE

(Only asked of those who have had any video-based visits with specialists in the past year, n=302)
QS3. On average, would you say your long-term residents' video-based visits with specialists provided better quality care than what can be done in person, worse quality care than what can be done in person, or about the same quality care?

Better quality	5
About the same quality	55
Worse quality	39
Refused	2

© 2026 SteelFisher GK et al. *JAMA Network Open.*

ADDITIONAL TIME BURDEN IMPOSED ON STAFF

(Only asked of those who have had any video-based visits with specialists in the past year, n=302)
QS4. Compared to in-person visits, how much additional time pressure would you say long-term residents' video-based visits with specialists placed on nursing staff, if any?

**A lot/A moderate amount (NET)**	**60**
A lot	16
A moderate amount	44
**A little/None at all (NET)**	**37**
A little	27
None at all	10
Refused	3

(Only asked of those who have had any video-based telemedicine visits with specialists in the past year, n=302)
Reasons for Telemedicine Specialist Visits for Long-Term Resident Care, Among SNFs who Reported Using Telemedicine Specialist Visits in the Past Year, n=302:

QS5. In your view, which of the following are reasons that your facility has used video-based visits for long-term residents' appointments with specialists in the past year?

	A reason (NET)	Major reason	Minor reason	Not a reason at all	Blank/ Missing
a. Helped improve communication between clinicians and other members of the care team	54	14	39	43	4
b. Helped prevent emergency room visits	52	22	31	44	4
c. Helped prevent admissions to the hospital	53	23	30	44	3
d. Helped prevent negative impacts on well-being of patients who have difficulty getting to and from appointments	77	45	32	20	3
e. Helped improve the quality of care for the patient at the moment	73	34	39	24	3
f. Were sometimes preferred by long-term residents over in-person visits	59	23	36	38	3
g. Were sometimes preferred by family members of long-term residents over in-person visits	53	14	39	45	3
h. Were sometimes preferred by SNF staff over in-person visits	38	11	27	59	3
i. Were sometimes preferred by specialists over in-person visits	66	26	40	31	3
j. Helped improve access to specialists after hours	45	19	27	51	3
k. Became a routine part of our practice	28	9	20	68	3

© 2026 SteelFisher GK et al. *JAMA Network Open.*

(Reported among those who reported "rarely" or "never" using video-based telemedicine visits with specialists in the past year, n=261)

Reasons for Telemedicine Disuse for Long-Term Resident Care, Among SNFs who Reported "Rarely" or "Never" Using Telemedicine in the Past Year, n=261:

QS6. In your view, which of the following are reasons your facility has NOT used [IF "rarely" used; "more"] video-based visits for long-term residents' appointments with specialists in the past year? Please indicate whether each of the following is a major reason, a minor reason or not a reason at all. Video-based visits with specialists…

	A reason (NET)	Major reason	Minor reason	Not a reason at all
a. Were not frequently offered by specialists	**73**	48	26	25
h. Did not offer much benefit in terms of quality of care over our normal practices	**62**	32	31	35
j. Did not seem to reduce emergency room visits or hospital admissions	**50**	20	30	47
c. Were not well liked by long-term residents	**49**	23	26	49
g. Took too much staff and nurse time	**49**	24	26	48
f. Were difficult to set up with all the technology	**46**	19	27	52
e. Were not well liked by family members of long-term residents	**45**	17	27	53
d. Were not well liked by staff and nurses	**41**	15	26	57
b. Were not well liked by specialists	**40**	17	23	57

© 2026 SteelFisher GK et al. *JAMA Network Open.*

(Reported among all DONs, n=379)
QS7. How often did long-term residents have video-based visits with specialists **[INSERT ITEM]**? *"Video-based" means the visit took place in the facility and had a video connection where the clinician could see and hear the resident live on a phone, tablet, laptop, or other computer.*

	**Always**	**Most of the time**	**About half the time**	**Less than half the time**	**Rarely**	**Never**	**Blank/ Missing**
a. during the peak of COVID	19	33	14	13	11	4	6
b. during the past year	1	3	4	19	51	18	3

(Only asked of those who have had any video-based telemedicine visits with specialists in the past year, n=302)
QS8. In the past year, who generally took on most of the responsibility for setting up video-based with specialists for long-term residents, meaning they did things like got the tablet or computer ready and logged into a portal?

Patients/family generally take on most of the responsibility	2
Our facility staff generally take on most of the responsibility	91
Patients/family and our facility staff generally take on the responsibility equally	6
Blank/Missing	2

(Reported among all DONs, n=379)
QS9. For your long-term residents, who generally takes on most of the responsibility of organizing in-person visits with specialists, meaning they make the appointment and arrange for transportation?

Patients/family generally take on most of the responsibility	1
Our facility staff generally take on most of the responsibility	91
Patients/family and our facility staff generally take on the responsibility equally	6
Blank/Missing	2

© 2026 SteelFisher GK et al. *JAMA Network Open.*

Methodological Details

Study Population and Survey Design

This survey was conducted online and by mail. Study participants were DONs or equivalent roles, as these key leaders are central decision makers for daily clinical operations of the SNF and have frontline visibility of the impact of telemedicine on patients and staff.

Data Collection

The survey was fielded February 7 – July 28, 2025, by SSRS (Glen Mills, PA). Survey participants were drawn from a list of 14,084 skilled nursing facilities (SNFs) in the U.S., based on data from the Centers for Medicare & Medicaid Services (CMS). 8/25/2026 2:05:00 PM From this list, we generated a stratified random sample of 3,100 SNFs, designed to ensure we included facilities of different sizes and varying telemedicine use, as measured by the number of Medicare fee-for-service part B visits in 2023 and the share of those visits delivered via telemedicine. To obtain accurate contact information, interviewers telephoned the list of 3,100 SNFs and successfully obtained information for 1,043 DONs, making multiple calls to each facility.

There were no major differences between the initial list of 3,100 and those for whom DON contact information was available with respect to the major demographic characteristics of the institutions.

Forty-two of these DONs were selected randomly and sent pilot surveys in Fall 2024; they were excluded from the final sample. The remaining 1,001 were divided into two randomized, replicate samples, which allowed us to evaluate the most effective recruiting strategies in the first round (January 2025) and implement those strategies in a second round (May 2025). Each wave of data collection included an initial invitation via FedEx, which included both a paper survey and return envelope as well as a URL for if the respondent wished to participate online. The invitations also included a $75 check as a non-contingent incentive for participation. Non-responders were sent multiple reminders via mail and email.

Survey Instrument and Measures

The questionnaire was developed by researchers at HSPH with consultation from the SSRS project team. Prior to programming the full questionnaire, SSRS researchers conducted two cognitive interviews with DONs via Zoom in Spring 2024, to examine how DONs would understand specific terms and questions. Phone interviewers called SNFs and attempted to identify the DON. Once identified, SSRS researchers would contact the DONs by email or phone to try to schedule a cognitive interview. Participants were offered a virtual gift card as thanks for participating. Questionnaires were edited for clarity and content based on these initial cognitive interviews.

Based on previous qualitative research by HSPH, it was determined that the Director of Nursing (DON) was the position within SNFs most able to answer the survey questions. To determine the DON in each sampled SNF, SSRS interviewers made multiple call attempts to each sampled SNFs to determine their full name and confirm contact information (phone number, mailing address and email address) for the DON. Interviewers were briefed on the importance of the project and the potential obstacles when attempting to verify the DON's contact information. Interviewers received detailed explanations as to why we were contacting DON, as well as strategies for addressing difficult gatekeepers and navigating phone systems.

© 2026 SteelFisher GK et al. *JAMA Network Open.*

After collecting the name and confirming the contact information of the DON, the interviewer also told the person on the phone that the DON should receive a FedEx packet in the mail in the coming months with a formal invitation to take part in the study. From this recruitment effort, 1,043 DONs were identified.

Forty-two records of these records were used in a pilot study described below and not included in the main data collection sample. These cases were used for a pilot test in Fall 2024. Based on the results of the pilot testing and feedback gathered from respondents, minor edits were made to the questionnaire and recruitment process.

Since the sample was based on a list of all SNFs in the US, all sampled SNFs were considered eligible. Based on the 1,001 invitation letters sent to the survey (Wave 1 release (n=400) and Wave 2 release (n=601), the survey cooperation rate is 39%.

Participants were asked about in-person and video visits for long-term residents in their SNF. Video visits were defined as those taking place in the facility with a video connection where the clinician could see and hear the resident live on a smartphone, tablet, laptop, or other computer.

Weighting and Statistical Analysis

To mitigate the risk of nonresponse bias and ensure the sample reflects the set of SNFs in the U.S., the sample was weighted to match the profile of the responding SNFs to the CMS's Long-Term Care Facility Characteristics[1]. Data were weighted by Census region, number of beds, 2023 visits, 2023 telemedicine rate, pandemic telemedicine visits, pandemic telemedicine rate, mean number of residents per day, provider type (Medicare, Medicaid, both), and SNFs' telemedicine use during COVID telemedicine (high/low) compared to their telemedicine use after COVID (high/low).

Comparisons between the survey sample and national benchmarks for key demographics are shown in the next section.

[1] https://data.cms.gov/quality-of-care/long-term-care-facility-characteristics

© 2026 SteelFisher GK et al. *JAMA Network Open.*

Demographic Characteristics of Skilled Nursing Facilities Surveyed and Comparison Between Sample and National Benchmarks

Data were weighted to represent the SNFs in the United States. The sample was calibrated to match SNF characteristics, taken from the CMS Skilled Nursing Facility Quality Reporting Program. eTable 1 below shows key characteristics of the sample compared to benchmark data.

Weights were trimmed to prevent individual interviews from having too much influence on final results. Differences between the weighted sample and the benchmarks are all within 1 percentage point.

© 2026 SteelFisher GK et al. *JAMA Network Open.*

eTable 1. Demographic Characteristics of Skilled Nursing Facilities Surveyed and Comparison Between Sample and National Benchmarks for Key Demographics

	n	Unweighted Percent	Weighted Percent	CMS (Benchmark) percent
**Region**				
Northeast	61	16.1%	16.8%	17.0%
Midwest	118	31.1%	31.2%	28.1%
South	138	36.4%	36.6%	36.6%
West	62	16.4%	15.4%	18.3%
**Number of Beds**				
<100	170	44.9%	48.0%	48.0%
100-199	184	48.5%	46.0%	45.9%
200+	25	6.6%	6.0%	6.1%
**Number of 2023 Visits**				
1st Quartile (lowest)	95	25.1%	27.1%	27.3%
2nd Quartile	95	25.1%	27.0%	27.3%
3rd Quartile	92	24.3%	23.4%	23.2%
4th Quartile (highest)	97	25.6%	22.4%	22.1%
**Telemedicine Rate (2023)**				
1st Quartile (lowest)	97	24.8%	26.1%	26.0%
2nd Quartile	81	22.2%	22.7%	22.6%
3rd Quartile	105	27.7%	26.7%	26.7%
4th Quartile (highest)	96	25.3%	24.4%	24.6%
**Pandemic Visits**				
1st Quartile (lowest)	98	25.9%	27.2%	27.4%
2nd Quartile	102	26.9%	26.5%	26.4%
3rd Quartile	86	22.7%	24.7%	24.8%
4th Quartile (highest)	93	24.5%	21.6%	21.3%
**Mean number of residents per day**				
<51	90	25.1%	27.1%	27.2%
51 – 75	101	26.4%	26.1%	26.0%
76 – 104	96	24.8%	25.1%	25.2%
>105	92	23.7%	21.7%	21.7%
**Insurance Coverage Type**				
Medicare and Medicaid	354	93.4%	94.8%	94.9%
Medicare only	20	5.3%	3.5%	3.5%
Medicaid only	5	1.3%	1.6%	1.6%
**Telemedicine use during/after COVID**				
High during Covid/Low after	101	26.6%	36.2%	36.5%
Low during Covid/high after	92	24.3%	21.9%	21.8%
High during Covid/high after	106	28.0%	27.0%	26.9%
Low during Covid/Low after	80	21.1%	14.9%	14.8%
**Pandemic telemedicine rate**				
<0.013	81	21.1%	19.2%	19.2%
0.013 – 0.03130673	84	23.0%	21.6%	21.7%
0.03130674 – 0.081	108	28.5%	26.9%	26.8%
>0.081	106	27.4%	32.4%	32.4%

Table notes: Authors' analysis of data from a 2025 nationally representative online and paper survey of 379 Directors of Nursing at U.S. Skilled Nursing Facilities.

Comparison Between the Eligible Sample and the Invited Sample

Researchers utilized a stratified random sample of n=3,100 SNFs from the CMS listing of all SNFs in the US. Institutions were identified only at the institutional level in the national database, so researchers reached out to all 3,100 organizations to gather the name and contact information for Directors of Nursing. All identifiable Directors of Nursing (n=1,043) were sent formal invitations to participate in the study.

Below shows a comparison of key characteristics between the initial list of 3,100 SNFs and those for whom DON contact information was available. There were no major differences between the initial list of 3,100 and those for whom DON contact information was available with respect to key characteristics of the institutions.

© 2026 SteelFisher GK et al. *JAMA Network Open.*

eTable 2. Comparison Between Invited Sample and Eligible Sample for Key Demographics

		Unweighted Percent Among Invited Sample n=1,043	Unweighted Percent Among Eligible Sample n=3,100
**Region**	Northeast	20.4%	18.8%
	Midwest	31.5%	30.2%
	South	32.5%	36.0%
	West	15.1%	15.0%
**Number of Beds**	<100	46.1%	45.8%
	100-199	48.2%	47.2%
	200+	5.7%	7.0%
**Number of 2023 Visits**	1st Quartile (lowest)	24.3%	25.4%
	2nd Quartile	24.8%	24.7%
	3rd Quartile	26.1%	24.8%
	4th Quartile (highest)	24.8%	25.1%
**Telemedicine Rate (2023)**	1st Quartile (lowest)	23.5%	25.0%
	2nd Quartile	25.2%	24.9%
	3rd Quartile	26.1%	24.8%
	4th Quartile (highest)	25.2%	25.4%
**Pandemic Visits**	1st Quartile (lowest)	24.0%	24.9%
	2nd Quartile	25.8%	25.0%
	3rd Quartile	25.6%	24.7%
	4th Quartile (highest)	24.6%	25.4%
**Mean number of residents per day**	<51	24.6%	24.9%
	51 – 75	26.3%	25.0%
	76 – 104	26.1%	25.2%
	>105	24.0%	25.0%
**Insurance Coverage Type**	Medicare and Medicaid	95.4%	95.0%
	Medicare only	3.4%	3.7%
	Medicaid only	1.2%	1.4%
**Telemedicine use during/after COVID**	High during Covid/Low after	23.4%	25.0%
	Low during Covid/high after	24.4%	25.0%
	High during Covid/high after	27.0%	25.0%
	Low during Covid/Low after	25.2%	25.0%
**Pandemic telemedicine rate**	<0.013	24.2%	25.1%
	0.013 – 0.03130673	23.9%	24.4%
	0.0310674 – 0.081	27.3%	25.1%
	>0.081	24.6%	25.4%

© 2026 SteelFisher GK et al. *JAMA Network Open.*
