# Supplementary material for: Perceptions of Telemedicine Use for Specialist Care in Skilled Nursing Facilities
Source: JAMA Netw Open. 2026 Oct 2;9(10):e2637194. doi: 10.1001/jamanetworkopen.2026.37194 (PMC13633543; doi:10.1001/jamanetworkopen.2026.37194)
Supplement: Supplementary file 2 [file jamanetwopen-e2637194-s002.pdf]

Data Sharing Statement

SteelFisher. Perceptions of Telemedicine Use for Specialist Care in Skilled Nursing Facilities. *JAMA Netw Open*. Published October 02, 2026. doi:10.1001/jamanetworkopen.2026.37194

Data
Data available: No

Additional Information
Explanation for why data not available: The authors are not making this dataset available because this data may be used for future publications.
